# Regional differences in the expression of tetrodotoxin-sensitive inward Ca^2+^ and outward Cs^+^/K^+^ currents in mouse and human ventricles

**DOI:** 10.1080/19336950.2019.1568146

**Published:** 2019-02-01

**Authors:** Wei Wang, Rebecca L. Mellor, Jeanne M. Nerbonne, C. William Balke

**Affiliations:** a Center for Cardiovascular Research, Department of Medicine, Cardiovascular Division, Washington University School of Medicine, St. Louis, MO, USA; b John Cochran Veterans Administration Medical Center, St. Louis, MO, USA

**Keywords:** Left ventricles (LV), interventricular septum, LV endocardium, LV epicardium, voltage-gated Na^+^ currents, *SCN5A*, Nav1.5

## Abstract

Tetrodotoxin (TTX) sensitive inward Ca^2+^ currents, *I*_Ca(TTX)_, have been identified in cardiac myocytes from several species, although it is unclear if *I*_Ca(TTX)_ is expressed in all cardiac cell types, and if *I*_Ca(TTX)_ reflects Ca^2+^ entry through the main, Nav1.5-encoded, cardiac Na^+^ (Nav) channels. To address these questions, recordings were obtained with 2 mm Ca^2+^ and 0 mm Na^+^ in the bath and 120 mm Cs^+^ in the pipettes from myocytes isolated from adult mouse interventricular septum (IVS), left ventricular (LV) endocardium, apex, and epicardium and from human LV endocardium and epicardium. On membrane depolarizations from a holding potential of −100 mV, *I*_Ca(TTX)_ was identified in mouse IVS and LV endocardial myocytes and in human LV endocardial myocytes, whereas only TTX-sensitive outward Cs^+^/K^+^ currents were observed in mouse LV apex and epicardial myocytes and human LV epicardial myocytes. The inward Ca^2+^, but not the outward Cs^+^/K^+^, currents were blocked by mm concentrations of MTSEA, a selective blocker of cardiac Nav1.5-encoded Na^+^ channels. In addition, in Nav1.5-expressing tsA-201 cells, *I*_Ca(TTX)_ was observed in 3 (of 20) cells, and TTX-sensitive outward Cs^+^/K^+^ currents were observed in the other (17) cells. The time- and voltage-dependent properties of the TTX-sensitive inward Ca^2+^ and outward Cs^+^/K^+^ currents recorded in Nav1.5-expressing tsA-201 were indistinguishable from native currents in mouse and human cardiac myocytes. Overall, the results presented here suggest marked regional, cell type-specific, differences in the relative ion selectivity, and likely the molecular architecture, of native *SCN5A-*/*Scn5a-* (Nav1.5-) encoded cardiac Na^+^ channels in mouse and human ventricles.

## Introduction

Inward calcium (Ca^2+^) currents that are sensitive to the voltage-gated sodium (Na^+^) channel toxin, tetrodotoxin (TTX), *I*_Ca(TTX),_ have been described in neuronal [1–3] and cardiac [4–7] cells. In the heart, *I*_Ca(TTX)_ has been reported to be present in human atrial myocytes [4] and in guinea-pig [5,8], rat [6,9–11], and mouse [12] ventricular myocytes. In addition to much lower amplitudes/densities than TTX-sensitive cardiac Na^+^ currents, *I*_Ca(TTX)_ displays slower activation and inactivation kinetics and apparent reversal potentials (values between −35 and +5 mV have been reported) that are more hyperpolarized than the reversal potentials (of +35 to +40 mV) typically reported for cardiac Na^+^ currents [4–6,8–11]. In some preparations, TTX-sensitive outward cesium (Cs^+^) currents have also been reported and suggested to underlie some of the variability in the amplitudes/densities and the measured reversal potentials of *I*_Ca(TTX)_ [1,6,9,10].

Although widely observed, the reported amplitudes/densities and detailed time- and voltage-dependent properties of the currents are quite heterogeneous, and it has been unclear if *I*_Ca(TTX)_ is a ubiquitous feature of the myocardium or, rather displays regional variations in expression, as observed with other cardiac ion channels, particularly voltage-gated K^+^ channels [13,14]. It has also been unclear if cardiac *I*_Ca(TTX)_ reflects inward Ca^2+^ flux through Na^+^ channels generated by the predominant cardiac voltage-gated Na^+^ channel pore-forming (α) subunit Nav1.5, encoded by *SCN5A/Scn5a*, or perhaps the expression and functioning of another Nav α subunit. Interestingly, in addition to Nav1.5 (*SCN5A/Scn5a*) several Nav α subunits, including Nav1.1 (*SCN1A/Scn1a*), Nav1.2 (*SCN2A/Scn2a*), Nav1.3 (*SCN3A/Scn3a*), Nav1.4 (*SCN4A/Scn4a*) and Nav1.6 (*SCN8A/Scn8a*) have been reported to be expressed in mammalian cardiac myocytes [15–18], albeit at much lower densities than Nav1.5. Nevertheless, these traditionally considered “non-cardiac” Nav α subunits are differentially expressed in the myocardium and have been suggested to play roles in the generation of cardiac Nav currents [15–19]. Previous studies focused on defining the molecular determinants of *I*_Ca(TTX)_, however, have provided conflicting results. It has, for example, been proposed that Nav1.5-encoded channels underlie *I*_Ca(TTX)_ [5,7,10]. In marked contrast, the results of experiments conducted on adult rat ventricular cells incubated with an antisense oligonucleotide directed against rat *Scn5a* have been interpreted as suggesting that a different (i.e. not Nav1.5) Nav α subunit underlies *I*_Ca(TTX)_ [20]. It has also been suggested, however, that voltage-gated cardiac Ca^2+^ channels, both high threshold, L-type Ca^2+^ channels [21–23] and low threshold, T-type Ca^2+^ channels [8,24], contribute to *I*_Ca(TTX)_.

In the experiments here, we used a combination of electrophysiological and pharmacological approaches to test the hypothesis that *I*_Ca(TTX)_ is differentially expressed in native (mouse and human) ventricular myocardium. These experiments revealed marked differences in the densities of *I*_Ca(TTX)_ and of TTX-sensitive outward Cs^+^/K^+^ currents in myocytes isolated from adult mouse interventricular septum (IVS), left ventricular (LV) endocardium, apex, and epicardium, as well as in human LV endocardial and epicardial myocytes. In addition, we present the results of experiments conducted on heterologously expressed human *SCN5A*- (Nav1.5-) encoded Na*^+^* channels that suggest that *both I*_Ca(TTX)_ *and* TTX-sensitive outward currents are generated by Nav1.5 (*SCN5A*/*Scn5a*) in mouse and human ventricular myocytes.

## Methods

### Ethical approvals

Animals were handled in accordance with the NIH Guide for the Care and Use of Laboratory Animals (NIH publication No. 85–23). All protocols involving animals were approved (Approval number 20140268) by the Animal Studies Committee at Washington University Medical School. Experiments were performed on adult (age range 9–15 week) male and female wild type (WT) C57BL6/J mice (Jackson Laboratory, Bar Harbor, ME).

Non-failing adult (age range 38–74 years; mean 59 ± 5 years) male (n = 5) and female (n = 2) human hearts, declined for transplantation, were obtained from Mid-America Transplant Services (St. Louis, MO). Approval (IRB ID# 201105210) for the use of human tissues was obtained from the Washington University in St. Louis Institutional Review Board (Washington University, St. Louis Department of Health & Human Services Federal Wide Assurance #FWA00002284), with a full HIPAA waiver.

### Isolation of mouse ventricular myocytes

Myocytes were isolated from adult (9–15-week-old) male and female C57BL6/J mice by enzymatic dissociation and mechanical dispersion using previously described methods [25,26]. Briefly, hearts were removed from anaesthetized (Avertin; 0.25 mg kg^−1^, I.P.; Sigma, St Louis, MO, USA) mice, mounted on a Langendorf apparatus, and perfused retrogradely through the aorta with 25 ml of a Ca^2+^-free Hepes-buffered Eagle’s balanced salt solution (Gibco/Invitrogen, Carlsbad, CA) supplemented with 6 mm glucose, amino acids and vitamins, followed by 25 ml of the same buffered solution containing (0.8 mg ml^−1^) Type II collagenase (Worthington Biochemical Corp., Lakewood, NJ) at 37°C for 15–20 min. Following the perfusion, the interventricular septum, left ventricular (LV) free wall and the LV apex were separated using a fine scalpel and iridectomy scissors; in some experiments, the endocardial and epicardial surfaces of the LV free wall were dissected. Tissue pieces were minced and incubated (separately) for 5 min in fresh enzyme-free buffer containing bovine serum albumin (5 mg ml^−1^; Sigma) and taurine (1.2 mg ml^−1^; Sigma) and subsequently dispersed by gentle trituration. The resulting cell suspensions were filtered. Cells were harvested by gravity sedimentation and resuspended in serum-free medium-199 (M-199; Sigma). Isolated myocytes were plated on laminin (Sigma) coated glass coverslips and maintained in a 95% air-5% CO_2_ incubator at 37°C for at least 1 h before using in electrophysiological experiments. Whole-cell recordings were obtained at room temperature (22 ~ 24°C) from mouse myocytes within 24 h of cell isolation.

### Isolation of human ventricular myocytes

Non-failing human hearts (*n *= 7; mean ± SEM age = 59 ± 5 years), deemed not suitable for transplantation for technical or non-cardiac reasons, were obtained from Mid-America Transplant Services). In each case, a transmural wedge of the left ventricle (LV), including a piece of the left anterior descending (LAD) artery, was excised. A surface branch of the LAD was cannulated and perfused with oxygenated Krebs buffer containing (in mm): 118 NaCl, 4.8 KCl, 1.2 MgCl_2_, 1.2 KH_2_PO_4_, 25 NaHCO_3_, 11 Glucose, 1.8 CaCl_2_, 0.01 Phenol Red, pH 7.3 at 37°C for 20 min. Wedges were subsequently perfused with Ca^2+^-free Krebs buffer supplemented with essential amino acids except L-glutamine (Gibco/Invitrogen), 1.5 nm insulin (Sigma), and 0.03 mm EGTA for ~15 min, before perfusion of supplemented Krebs buffer containing 35 µM CaCl_2_ and 0.5–0.7 mg/ml of Type II collagenase (Worthington) at 37°C. Following 20 min perfusion of the enzyme-containing solution, mayo scissors were used to remove the epicardial (LV epi) and endocardial (LV endo) regions of the LV free wall; these were minced and incubated (separately) in fresh collagenase-containing solution for an additional 15 min at 37°C. Following trituration, the resulting cell suspensions were filtered and resuspended in serum-free M-199 (Sigma). Isolated myocytes were plated on laminin-coated coverslips and maintained in a 95% air-5%CO_2_ incubator at 37°C for at least 1 h before using in electrophysiological experiments. Whole-cell recordings were obtained at room temperature (22 ~ 24°C) from human LV myocytes within 24 h of cell isolation.

### Heterologous expression of human Nav1.5

tsA-201 cells, obtained from the American Tissue Culture Collection (Manassas, VA), were maintained in Dulbecco’s Modified Eagle Medium (Gibco/Invitrogen), supplemented with 5% horse serum (Gibco/Invitrogen), 5% heat-inactivated fetal calf serum (Gibco/Invitrogen) and 1 unit/ml penicillin-streptomycin (Gibco/Invitrogen), in a 95% air-5%CO_2_ incubator at 37°C. Cells were passaged at confluence every 2–3 days by brief trypsinization. For electrophysiological recordings, tsA-201 cells were plated on 35 mm tissue culture (plastic) dishes at low density and transfected *in situ*. For transfections, 0.2 *µ*g of an enhanced green fluorescent protein- (eGFP-) expressing plasmid alone, or 2 *µ*g of a human *SCN5A*- (Nav1.5-) expressing and 0.2 *µ*g of the eGFP-expressing plasmid, was mixed with 2 *µ*g Lipofectamine 2000 (Life Technologies Inc., Gaithersburg, MD) in Opti-MEM (Gibco/Invitrogen), incubated at room temperature for ~30 min, and subsequently added to the tissue culture dishes containing tsA-201 cells, maintained in a 95% air-5%CO_2_ incubator at 37°C. Approximately 8 h later, the plasmid-containing solutions were replaced with normal cell culture medium (see above). eGFP expression was identified under epifluorescence illumination 24–36 h after transfections, and electrophysiological recordings were obtained from eGFP-expressing cells.

### Electrophysiological recordings

Whole-cell voltage-clamp recordings were obtained at room temperature (22–24°C) from mouse and human ventricular myocytes within 24 hr of isolation and from transfected tsA-201 cells using a Dagan 3900A (Dagan Corporation) amplifier, interfaced to a Digidata 1332A A/D converter (Molecular Devices) and pClamp 10.3 (Molecular Devices). Recording pipettes routinely contained (in mmolL^−1^): 120 glutamic acid, 120 CsOH, 10 HEPES, 0.33 MgCl_2_, 20 tetraethylammonium chloride (TEA-Cl), 4 Mg-ATP, and 5 EGTA (pH adjusted to 7.3 with CsOH) [6]. In some experiments, N-methyl-D-glucamine (NMDG^+^) was used in place of the Cs^+^ (to eliminate the outward currents through TTX-sensitive channels; the pipette solution contained (in mM): 150 NMDG^+^, 4 Mg-ATP and 5 EGTA (pH adjusted to 7.3 with HCl/Tris base). In experiments focused on recording outward K^+^ currents, pipettes contained (in mmolL^−1^): 135 KCl, 10 EGTA, 10 HEPES, 5 glucose, 5 K_2_ATP (pH adjusted to 7.2 with KOH) [27]. Pipette resistances were 1.5–2.0 MΩ when filled with either recording solution. In all experiments, junction potentials were compensated prior to seal formation.

Electrophysiological data were acquired at 10–20 kHz and signals were low-pass filtered at 5 kHz before digitization and storage. After the formation of a giga-seal (> 1 GΩ) and establishment of the whole-cell configuration, brief (10 ms) ± 10 mV voltage steps from a holding potential of −70 mV were presented to allow measurements of whole-cell membrane capacitances (C_m_), input resistances (R_in_) and series resistances (R_s_). The mean ± SEM C_m_ and R_in_ determined for mouse ventricular myocytes were 156 ± 6 *p*F and 997 ± 184 MΩ (n = 94) and, for human ventricular myocytes, were 162 ± 20 *p*F and 1354 ± 309 MΩ (n = 26). In each cell, C_m_ and R_s_ were compensated by ≥ 85%; voltage errors resulting from uncompensated series resistance were always <2 mV and were not corrected. Leak currents were always < 50 *p*A and were not corrected.

Cells were initially superfused (rate 2–3 ml min^−1^) with a 0 mm Na^+^ bath solution containing (in mmolL^−1^): 140 TEA-Cl, 10 CsCl, 1 MgCl_2_, 2 CaCl_2_, 10 HEPES and 10 glucose (pH adjusted to 7.4 with CsOH) [6]. In some experiments, NMDG^+^ replaced the TEA-Cl in the bath solution; the NMDG^+^ bath solution contained (in mM): 150 NMDG+, 1 MgCl_2_, 2 CaCl_2_, 10 HEPES and 10 glucose (pH adjusted to 7.4 with HCl/Tris base). Whole-cell inward and outward currents, evoked in response to 300 ms voltage steps to potentials between −90 and +25 mV from a HP of −50 mV and −100 mV, were recorded; depolarizing voltage steps were presented in 5 mV increments at 2 s intervals. A multi-manifold perfusion system [27] was used to apply bath solutions of varying Na^+^ and/or Ca^2+^ concentrations, as well as the Tetrodotoxin- (TTX-; Alomone labs, Jerusalem), the Ni^2+^- (NiCl_2_; Sigma), the verapamil- (verapamil hydrochloride; Sigma) and the 2-aminoethyl methanethiosulfonate hydrochloride- (MTSEA-; MTSEA-Cl (US Biologicals, San Diego, CA) containing bath solutions during recordings.

MTSEA-Cl was dissolved in dimethyl sulfoxide (DMSO) and diluted in bath solution to a final concentration of 2 mm; control experiments revealed that the DMSO (0.1%) in the MTSEA-containing bath solution did not measurably affect the passive membrane properties of myocytes or the amplitudes/properties of voltage-dependent currents. The waveforms of the TTX-sensitive currents were obtained by off-line digital subtraction of records obtained in the presence of TTX from the controls.

### Data analysis and statistics

Electrophysiological data were compiled and analyzed using Clampfit 10.3 (Molecular Devices) and GraphPad (Prism). Peak inward/outward current amplitudes were measured in each cell at various test potentials and normalized to the whole-cell membrane capacitance (in the same cell); current densities (pA/pF) are reported. The decay phases of currents (*I*_Ca(TTX)_, *I*_Na_ and *I*_Ca.L_) were fitted by one (y(t) = A*exp(-t/*Ƭ*) + B) exponential (for *I*_Ca(TTX)_) or two (y(t) = A_fast_*exp(-t/*Ƭ*_fast_) + A_slow_*exp(-t/*Ƭ*_slow_) + B) exponentials (for *I*_Na_ and *I*_Ca.L_), where *Ƭ, Ƭ*_fast_ and *Ƭ*_slow_ are the decay time constants, A, A_fast_ and A_slow_ are the amplitudes of the inactivating current components, and, in each case, B corresponds to the steady-state component of the total current. The voltage-dependences of activation of the peak inward currents through Nav1.5-encoded channels were determined by first measuring the peak amplitudes of the currents evoked at various test potentials from a holding potential of −100 mV. Conductances (G_Na_) were calculated and normalized to the maximal conductance (G_Na,max_) determined in the same cell. Mean ± SEM normalized conductances (G_Na/_G_Na,max_) were then plotted as a function of the test potential and fitted with a Boltzmann equation: G_Na_ = G_Na,max_/[1+ exp(V_1/2_-V)/*k*], where G_Na,max_ is the maximal conductance, V is the test potential, V_1/2_ is the potential of half maximal activation, and *k* is the slope factor. All data are presented as means ± SEM. The statistical significance of observed differences was evaluated using a paired (compared in the same cell) two-tailed student’s t test, one-way or two-way ANOVA, as indicated in the text or figure legends; *P* values ≤ 0.05 were considered statistically significant.

## Results

### Ttx-sensitive inward Ca^2+^ currents in mouse interventricular septum myocytes

**Figure 1.**
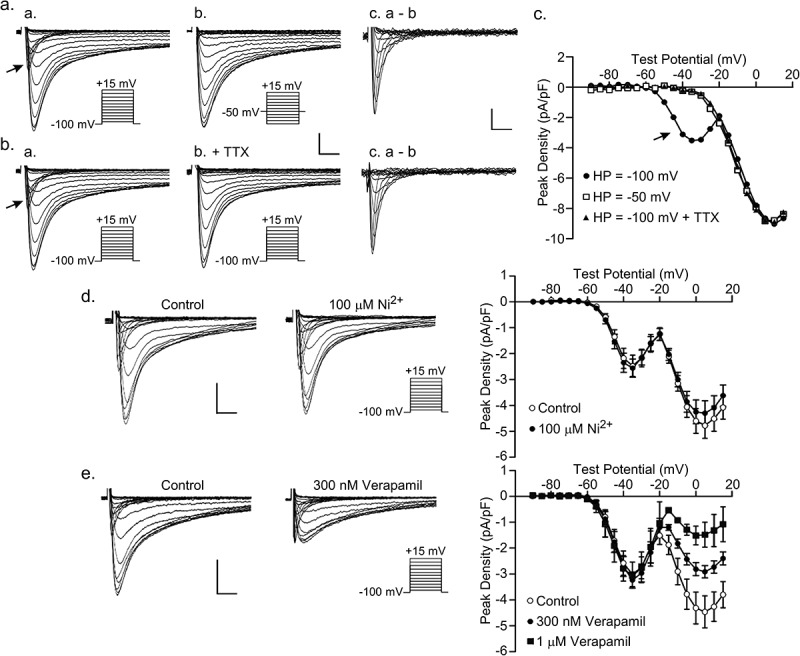
**TTX-sensitive Ca^2+^ currents in mouse interventricular septum (IVS) myocytes**. Voltage-gated **i**nward Ca^2+^ currents (*I*_Ca_) in mouse IVS myocytes were measured with 2 mm Ca^2+^ and 0 mm Na^+^ in the bath (see Methods) and 120 mm Cs^+^ in the recording pipettes. (a) Representative *I*_Ca_, elicited in an IVS myocyte in response to 300 ms voltage steps to test potentials between −90 and +15 mV (in 5 mV increments) from a holding potential (HP) of −100 mV, are shown in panel a; currents evoked in the same cell from a HP of −50 mV are shown in panel b. The rapidly activating and inactivating component of *I*_Ca_ (arrow in panel a) is not evident in the records evoked from −50 mV (panel b). The waveforms of this component, obtained by off-line digital subtraction of the currents evoked at test potentials between −90 mV and −25 mV from a HP of −50 mV from those evoked from a HP of −100 mV, are shown in panel c. (b) In the same cell as in (a), currents evoked from −100 mV were recorded before (panel a) and after (panel b) the addition of 10 *µ*m TTX to the bath. The waveforms of the TTX-sensitive Ca^2+^ current (*I*_Ca(TTX)_), obtained by off-line digital subtraction of the currents recorded at test potentials between −90 mV and −25 mV in the absence (panel a) and presence (panel b) of TTX, are shown in panel c. In (a) and (b), the scale bars are 2 pA/pF and 10 ms in panels a and b, and 1pA/pF and 10 ms in panel c. (c) Peak inward Ca^+^ currents evoked from −100 mV in the absence (●) and the presence (▲) of TTX and from −50 mV (□) are plotted as a function of the test potential; the arrow indicates the TTX-sensitive inward Ca^+^ current, *I*_Ca(TTX)_. (d) Representative *I*_Ca_, elicited in an IVS myocyte using the same voltage-clamp protocol (illustrated adjacent to the current records) as in (b), before and after exposure to the 100 *µ*m Ni^2+^-containing bath solution; scale bars are 2 pA/pF and 10 ms. Similar results were obtained on 6 cells. Mean ± peak inward Ca^+^ current densities evoked from −100 mV before (o) and after (●) the application of the 100 *µ*m Ni^2+^-containing bath solution are plotted as a function of the test potential in the panel on the right. (*E*) Representative *I*_Ca_, elicited in an IVS myocyte before (o) and after (●) the application of the 300 *n*m verapamil-containing bath solution; scale bars are 2 pA/pF and 10 ms. Similar results were obtained on 4 cells. Mean ± peak inward Ca^+^ current densities evoked from −100 mV in control bath (o) and following exposure to the 300 *n*m (●) or the 1 *µ*m (■) verapamil-containing bath solution are plotted as a function of the test potential in the panel on the right.

Whole-cell voltage-clamp recordings, obtained from isolated mouse interventricular septum (IVS) myocytes with 2 mm Ca^2+^ and 0 mm Na^+^ in the bath solution and 120 mm Cs^+^ in the recording pipettes, revealed two distinct inward current components. As illustrated in the representative recordings presented in Figure 1(a) (panel a), a rapidly activating and inactivating inward current (arrow in panel a) was observed on membrane depolarizations from a holding potential (HP) of −100 mV to the more hyperpolarized (e.g. −55 mV to −25 mV) test potentials, followed by a more slowly activating and inactivating current at the more depolarized membrane potentials. Only the more slowly activating/inactivating current was observed when the currents were evoked from a HP of −50 mV (Figure 1(a), panel b), consistent with the presence of a high threshold voltage-gated inward cardiac Ca^2+^ current [28–30], now typically referred to as *I*_Ca_ “long lasting” or *I*_Ca,L_ [23,31]. The rapidly activating and inactivating component of the inward Ca^2+^ current was isolated by off-line digital subtraction of the currents evoked from −50 mV from those evoked from −100 mV (Figure 1(a), panel c). Peak inward Ca^2+^ current densities evoked from −100 mV (●) and −50 mV (□) in this cell, are plotted as a function of the test potential in Figure 1(c). The current component seen only on depolarizations from a HP of −100 mV (●) is indicated by the arrow.

Additional experiments revealed that the rapidly activating and inactivating component of the inward Ca^2+^ current in mouse IVS myocytes is selectively blocked (Figure 1(b)) by *μ*M concentrations of the Na^+^ channel selective toxin, tetrodotoxin (TTX). As illustrated in Figure 1(b), panel b, bath application of 10 *μ*m TTX eliminated the low threshold, rapidly activating and inactivating component of the inward Ca^2+^ current without measurably affecting the slowly activating and inactivating Ca^2+^ current, *I*_Ca,L._ The current-voltage relation for *I*_Ca,L_ (Figure 1(b), panel b), evoked from −100 mV in the presence of 10 *μ*m TTX (▲), is plotted in *C*. Offline digital subtraction of the inward currents evoked from a HP of −100 mV in the presence of TTX (Figure 1(b), panel b), from those recorded in the absence of TTX (Figure 1(b), panel a) revealed the TTX-sensitive component of *I*_Ca_ (Figure 1(b), panel c), similar to TTX-sensitive inward Ca^2+^ currents previously described in cardiac myocytes from several species that have been referred to as *I*_Ca(TTX)_ [5–7,9,10,12,20].

Although low threshold, “T” type, Ca^2+^ channels [32] have previously been identified in mammalian cardiac myocytes [29], additional experiments revealed that the rapidly activating and inactivating inward Ca^2+^ current component evoked from a holding potential of −100 mV in isolated mouse IVS myocytes is not affected by the selective T-type Ca^2+^ channel blocker Ni^2+^ (Figure 1(d)) at 100 µM, suggesting that the TTX-sensitive inward Ca^2+^ current does not reflect Ca^2+^ entry through T-type Ca^2+^ channels. In addition, although the high threshold, slowly activating and inactivating Ca^2+^ current component is reduced by the selective L-type Ca^2+^ channel blocker verapamil [33] in a dose-dependent manner (Figure 1(e)), verapamil does not measurably affect the low threshold, rapidly activating and inactivating TTX-sensitive component of the inward Ca^2+^ current that we refer to as *I*_Ca(TTX)_.

**Figure 2.**
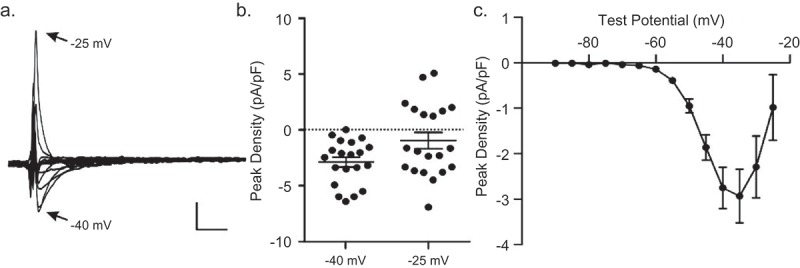
**Heterogeneous expression of *I*_Ca(TTX)_ in mouse IVS myocytes**. Whole-cell currents, elicited in response to 300 ms voltage steps to test potentials between −90 and −25 mV (in 5 mV increments) from a HP of −100 mV, were recorded from mouse IVS myocytes (n = 20) with 2 mm Ca^2+^/0 mm Na^+^ in the bath and 120 mm Cs^+^ in the pipettes before and after application of 10 *µ*m TTX, as described in the legend to Figure 1. (a) The waveforms of the TTX-sensitive currents, obtained by off-line digital subtraction of the records in the absence and presence of 10 *µ*m TTX, in one of these IVS cells is shown; the scale bars are 1 pA/pF and 10 ms. The currents evoked at test potentials of −40 mV (●) and −25 mV (●) are indicated. (b) The peak current densities at −40 mV and −25 mV in individual IVS cells (n = 14) are plotted. (c) Mean ± SEM (●; n = 20) peak TTX-sensitive current densities in mouse IVS cells are plotted as a function of test potential; the mean ± SEM extrapolated reversal potential of the TTX-sensitive currents in mouse IVS myocytes was −18.1 ± 3.7 mV.

Using the protocol illustrated in Figure 1 B, *I*_Ca(TTX)_ was identified in all mouse IVS myocytes (n = 20) examined, although there was, as is illustrated in Figure 2, considerable variability in peak inward current amplitudes/densities among cells (Figure 2(b)). In addition, in 8 of the 20 IVS cells studied using this protocol, outward (Cs^+^) currents were observed at −25 mV (Figure 2(b)). The mean ± SEM peak TTX-sensitive current densities measured from recordings obtained from mouse IVS cells (n = 20) during voltage-steps to potentials between −90 and −25 mV (in 5 mV increments) are plotted as a function of test potential in Figure 2(c); at more positive (≥ −20 mV) test potentials, the mean ± SEM peak currents were outward.

### *I*_ca(ttx)_ is blocked by MTSEA, a selective antagonist of Nav1.5-encoded Na^+^ channels

**Figure 3.**
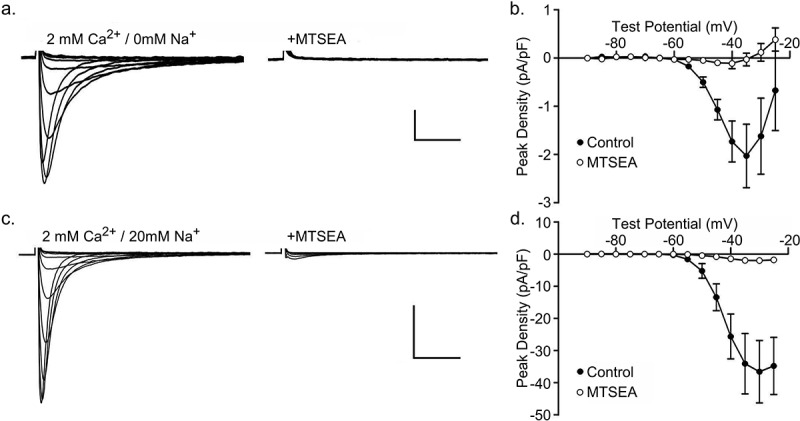
**MTSEA blocks TTX-sensitive inward Ca^2+^ currents in mouse IVS myocytes**. (a) With 2 mm Ca^2+^/0 mm Na^+^ in the bath and 120 mm Cs^+^ in the pipette, ***I*_Ca(TTX)_** was recorded in a mouse IVS cell as described in the legend to Figure 2. On application of 2 mm MTSEA, the currents were eliminated; scale bars are 1 pA/pF and 10 ms. (b) Mean ± SEM peak current densities measured in mouse IVS myocytes (n = 14) before (●) and after (o) application of the MTSEA-containing bath solution are plotted as a function of the test potential. (c) Robust inward Na^+^ currents (*I*_Na_) were recorded from isolated mouse IVS myocytes with bath solution containing 2 mm Ca^2+^/20 mm Na^+^ and 120 mm Cs^+^ in the pipette. Similar to **of *I*_Ca(TTX)_** (a), *I*_Na_ was also eliminated on perfusion of 2 mm MTSEA-containing bath solution (c). Scale bars are 10 pA/pF and 10 ms. (*D*) Mean ± SEM (n = 4) peak current densities in IVS myocytes with 2 mm Ca^2+^/20 mm Na^+^ in the bath before (●) and after (o) MTSEA are plotted as a function of the test potential.

Addition of (2 mm) MTSEA, a selective inhibitor of Nav1.5-encoded cardiac Na^+^ channels [19,34,35] to the bath solution eliminated *I*_Ca(TTX)_ in mouse IVS myocytes. As illustrated in the representative recordings obtained from a mouse IVS cell presented in Figure 3(a), inward Ca^2+^ currents were recorded under control conditions with 2 mm Ca^2+^and 0 mm Na^+^ in the bath and 120 mm Cs^+^ in the recording pipette (left panel). When the bath solution was switched to one containing 2 mm MTSEA, the currents were eliminated (Figure 3(a), right panel). Similar results were obtained in experiments on 14 adult mouse IVS myocytes and the mean ± SEM (n = 14) current-voltage relations determined for these cells before (●) and after (o) exposure to 2 mm MTSEA are plotted in Figure 3(b); no inward or outward currents were recorded in the presence of 2 mM MTSEA.

When recordings were obtained from isolated adult mouse IVS myocytes with increased (from 0 mm to 20 mm) Na^+^ in the bath, much larger amplitude inward (Na^+^) currents were observed (Figure 3(c), left panel); note the different scale bar in Figure 3(c), compared with Figure 3(a). Application of 2 mm MTSEA also eliminated the inward Na^+^ currents (Figure 3(c), right panel). The mean ± SEM (n = 4) current-voltage relations for the peak currents recorded in isolated mouse IVS cells with 20 mm Na^+^ and 2 mm Ca^2+^ in the bath, before (●) and after (o) the application of 2 mm MTSEA are presented in Figure 3(d).

### Regional differences in expression of TTX-sensitive inward Ca^2+^ and outward Cs^+^ currents in adult mouse ventricles

**Figure 4.**
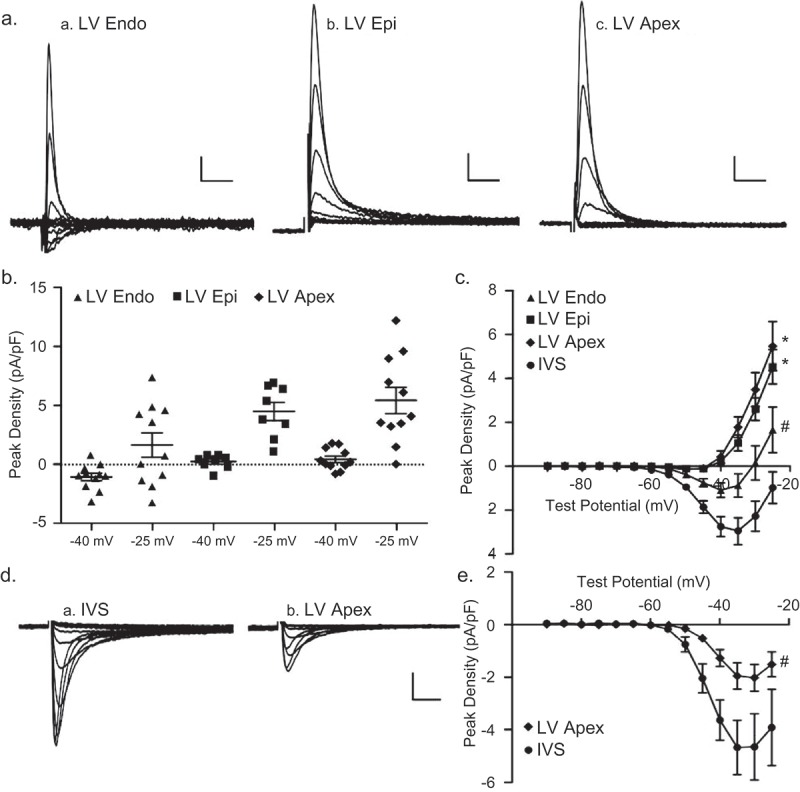
**Regional differences in TTX-sensitive currents in adult mouse ventricles**. TTX-sensitive currents were recorded and isolated as described in the legend to Figure 2 from myocytes isolated from mouse LV endocardium (LV Endo), LV epicardium (LV Epi) and LV apex. (a) Representative TTX-sensitive currents in LV Endo (panel a), LV Epi (panel b) and LV Apex (panel c) myocytes, are shown; scale bars are 0.5 pA/pF and 10 ms in panel a and 1 pA/pF and 10 ms in panels b and c. Small inward Ca^2+^ currents were seen at the more hyperpolarized test potentials in LV Endo myocytes, and outward Cs^+^ currents were recorded at the more positive test potentials. Only TTX-sensitive outward Cs^+^ currents were observed in LV Epi and LV Apex myocytes. (b) Peak densities of the TTX-sensitive currents measured at −40 mV and −25 mV in individual LV Endo (▲; n = 11), LV Epi (■; n = 8) and LV apex (♦; n = 11) myocytes are plotted. (c) Mean ± SEM peak TTX-sensitive current densities measured in LV Endo (▲; n = 11), LV Epi (■; n = 8) and LV apex (♦; n = 11) myocytes are plotted as a function of the test potential; the mean ± SEM peak TTX-sensitive current densities in adult mouse IVS myocytes (see Figure 2(c)) are replotted here (●) to facilitate direct comparisons of the currents across cell types. ^*,‡^ Current-voltage plots in LV Apex, LV Epi and LV Endo myocytes are significantly (two-way ANOVA) different from the current-voltage plot for the TTX-sensitive currents in IVS myocytes (●) at the *P < 0.001 and ^‡^*P* < 0.05 levels. (d) TTX-sensitive inward Ca^2+^ currents were recorded from IVS (panel a) and LV apex (panel b) myocytes with the NMDG^+^-containing pipette solution (see: **Methods**); scale bars are 2 pA/pF and 10 ms. (*E*) Mean ± SEM peak TTX-sensitive inward Ca^2+^ current densities measured in IVS (●; n = 4) and LV apex (♦; n = 8) myocytes with the NMDG^+^-containing pipette solution (see: **Methods**) are plotted as a function of the test potential. Although the current-voltage plots are similar, the amplitudes/densities of the inward Ca^2+^ currents are lower (^‡^*P* < 0.05; two-way ANOVA) in mouse LV apex, compared with IVS, myocytes at all test potentials.

Additional experiments were completed to quantify the expression of TTX-sensitive inward/outward currents in myocytes isolated from different regions of adult mouse left ventricles (LV). As in the experiments on mouse IVS myocytes, voltage-activated inward/outward currents, evoked from a HP of −100 mV in response to 300 ms test potentials between −90 mV to −25 mV (in 5 mV increments) were recorded with 2 mm Ca^2+^/0 mm Na^+^ in the bath and 120 mm Cs^+^ in the recording pipettes, before and after the addition of 10 µM TTX. Representative recordings of the TTX-sensitive currents obtained from myocytes isolated from the LV endocardium (LV Endo, panel a), LV epicardium (LV Epi, panel b) and LV apex (LV Apex, panel c) are presented in Figure 4(a); note that the scale bars are different in panels a (0.5 pA/pF), b and c (1.0 pA/pF). The peak TTX-sensitive inward/outward current densities measured in individual adult mouse LV Endo (▲; n = 11), LV Epi (■; n = 8) and LV apex (♦; n = 11) myocytes at test potentials of −40 mV and −25 mV are presented in Figure 4(b)). Mean ± SEM peak TTX-sensitive current densities in adult mouse LV Endo (▲; n = 11), LV Epi (■; n = 8) and LV apex (♦; n = 11) myocytes are plotted as a function of test potential in Figure 4(c).

Although prominent outward Cs^+^ currents were recorded in cells from all three regions of the adult mouse LV, outward currents were larger in LV epi and LV apex myocytes, and inward *I*_Ca(TTX)_ was *only* evident in recordings from LV endo cells (Figure 4(a), panel a). The mean ± SEM peak *I*_Ca(TTX)_ density (at −40 mV) in LV endo cells of −0.9 ± 0.5 *p*A/*p*F (n = 11), however, was much lower than the mean + SEM peak *I*_Ca(TTX)_ density (at −40 mV) of (−2.9 ± 0.5 *p*A/*p*F (n = 20) measured in IVS myocytes. Similar results were obtained when 120 mm K^+^ replaced the Cs^+^ in the recording pipettes (data not shown). These combined observations suggested the interesting hypothesis and that inward TTX-sensitive Ca^2+^ currents might be revealed in mouse LV apex (and LV epi) myocytes if the outward Cs^+^/K^+^ currents were blocked. To test this hypothesis, additional experiments were conducted with NMDG^+^, which does not permeate voltage-gated Na^+^ channels [10], used in place of the Cs^+^/K^+^, in the recording pipettes. As illustrated in Figure 4(d), TTX-sensitive inward Ca^2+^ currents were recorded from LV apex and, as expected, from IVS myocytes with NMDG^+^-containing pipette solution (see: **Methods**). In addition, although the mean ± SEM amplitudes/densities of the currents are different, the voltage-dependent properties of *I*_Ca(TTX)_ recorded in IVS and LV apex myocytes with the NMDG^+^-containing pipette solution are indistinguishable (Figure 4(e)).

### Ionic dependence of TTX-sensitive inward currents in mouse IVS myocytes

**Figure 5.**
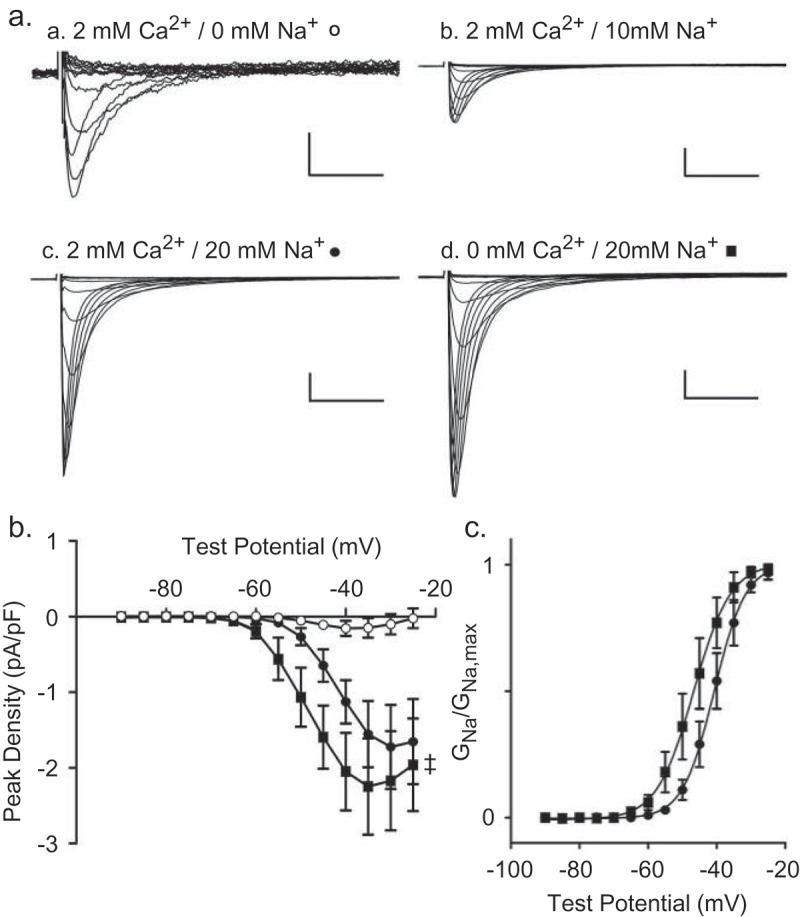
**Extracellular Ca^2+^ modulates the voltage-dependence of Na^+^ current activation in mouse IVS myocytes**. Inward currents, evoked from a HP of −100 mV and from a HP of −50 mV, were recorded in mouse IVS myocytes with 2 mm Ca^2+^/0 mm Na^+^ in the bath and 120 mm Cs^+^ in the recording pipettes as described in the legend to Figure 1. The bath solution was then changed sequentially to one containing 2 mm Ca^2+^/10 mm Na^+^, 2 mm Ca^2+^/20 mm Na^+^ and 0 mm Ca^2+^/20 mm Na^+^ and the currents were again recorded (from both HPs). (a) Representative inward current waveforms recorded under these different ionic conditions, obtained by off-line digital subtraction of the currents evoked from −50 mV from those evoked from −100 mV, are illustrated. Increasing the Na^+^ concentration in the bath from 0 mm (panel a; o) to 10 mm (panel b), and further to 20 mm (panel c; ●) increased inward current amplitudes. Exchange of the extracellular solution to one containing 0 mm Ca^2+^ (replaced by 2 mm Mg^2+^) and 20 mm Na^+^ revealed in a further increase in the peak inward current amplitude (panel d; ■). Scale bars are 1 pA/pF and 10 ms in panel and 10 pA/pf and 10 ms in panels b, c and d. (b) Similar results were obtained in 4 cells, and mean ± SEM peak inward current densities under the different recording conditions (o,●,■) are plotted as a function of the test potential. The current-voltage relation was shifted (^‡^*P* < 0.01; two way ANOVA) for the currents recorded with 0 mm Ca^2+^/20 mm Na^+^ (■) in the bath, compared with the currents recorded with 2 mm Ca^2+^/20 mm Na^+^ (●) in the bath. (c) Mean ± SEM (n = 4) normalized voltage-dependences of activation of the peak Na^+^ conductance for the currents recorded in 20 mm Na^+^ and 2 mm Ca^2+^ (●) and 20 mm Na^+^ and 0 Ca^2+^ (■) are plotted as a function of test potential and fitted with single Boltzmanns. The *V*_1/2_ of current activation was shifted (^‡^*P *< 0.01; paired Student’s *t* test) to −46 ± 3 mV (*k* = 4.3 ± 0.3), with 0 mm Ca^2+^/20 mm Na^+^) (■) in the bath, from −40 ± 2 mV (*k *= 4.1 ± 0.5), with 2 mm Ca^2+^/20 mm Na^+^ (●) in the bath.

Additional experiments were conducted to compare the effects of extracellular Ca^2+^ and Na^+^ on the time and voltage-dependent properties of TTX-sensitive inward currents in mouse IVS myocytes. Currents, evoked during voltage steps to test potentials between −90 mV and −25 mV from a HP of −100 mV and from a HP of −50 mV, were recorded in isolated adult mouse IVS myocytes with 2 mm Ca^2+^/0 mm Na^+^ in the bath and 120 mm Cs^+^ in the recording pipettes. The bath solution was then changed sequentially to one containing 2 mm Ca^2+^/10 mm Na^+^, followed by 2 mm Ca^2+^/20 mm Na^+^ and, finally, 0 mm Ca^2+^/20 mm Na^+^, and the voltage-clamp paradigms were repeated. The rapidly activating and inactivating inward currents under each recording condition, isolated by off-line digital subtraction of the currents evoked from −50 mV from those evoked from −100 mV, are presented in Figure 5(a) (panels a-d). As illustrated, the inward current amplitude increased markedly (note the change in the scale) with the addition of 10 mm Na^+^ (and 2 mm Ca^2+^) to the bath (Figure 5(a), panel b). The time- and voltage-dependent properties of the inward currents, however, were similar to those observed with 0 Na^+^/2 mm Ca^2+^ in the bath (Figure 5(a), panel a).

Increasing the Na^+^ in the bath to 20 mm (in the presence of 2 mm Ca^2+^) further increased the peak inward current (Figure 5(a), panel c). With the removal of the Ca^2+^ (replaced with 2 mm Mg^2+^) from the bath solution, inward current amplitudes were increased (Figure 5(a), panel d), compared with the currents recorded with 2 mm Ca^2+^/20 mm Na^+^ (Figure 5(a), panel c). The mean ± SEM (n = 4) peak inward current versus voltage relations for the records obtained with 2 mm Ca^2+^/0 mm Na^+^ (°), 2 mm Ca^2+^/20 mm Na^+^ (●) and 0 mm Ca^2+^/20 mm Na^+^ (■) in the bath were determined and are presented in Figure 5(b). In addition to the increase in current amplitude/density, there is a marked leftward shift in the peak current-voltage plot for the records obtained with 0 mm Ca^2+^/20 mm Na^+^ (■), compared with 2 mm Ca^2+^/20 mm Na^+^ (●). The normalized voltage-dependences of activation of the inward currents recorded with 2 mm Ca^2+^/20 mm Na^+^ (•) and 0 mm Ca^2+^/20 mm Na^+^ (■) in the bath, each fitted with a single Boltzmann, are presented in Figure 5(c).

### TTX-sensitive inward Ca^2+^ and outward Cs^+^/k^+^ currents in tsA-201 cells expressing *SCN5A*

Additional experiments were conducted to detail the properties of the TTX-sensitive currents expressed in tsA-201 cells transiently transfected with a plasmid encoding human *SCN5A* (Nav1.5) and enhanced green fluorescent protein (eGFP). Using voltage-clamp paradigms similar to those used in the experiments on mouse ventricular myocytes described above, currents, evoked during voltage steps to test potentials between −90 mV and −25 mV from a HP of −100 mV and from a HP of −50 mV, were recorded from eGFP-positive tsA-201 cells with 2 mm Ca^2+^/0 mm Na^+^ in the bath and 120 mm Cs^+^ or 145 mm K^+^ in the recording pipettes. The bath solution was then changed sequentially to 2 mm Ca^2+^/10 mm Na^+^, followed by 2 mm Ca^2+^/20 mm Na^+^ and, finally, 0 mm Ca^2+^/20 mm Na^+^, and the voltage-clamp paradigms were repeated. The currents obtained with offline digital subtraction of the currents evoked from −50 mV from those evoked from −100 mV were analyzed.

**Figure 6.**
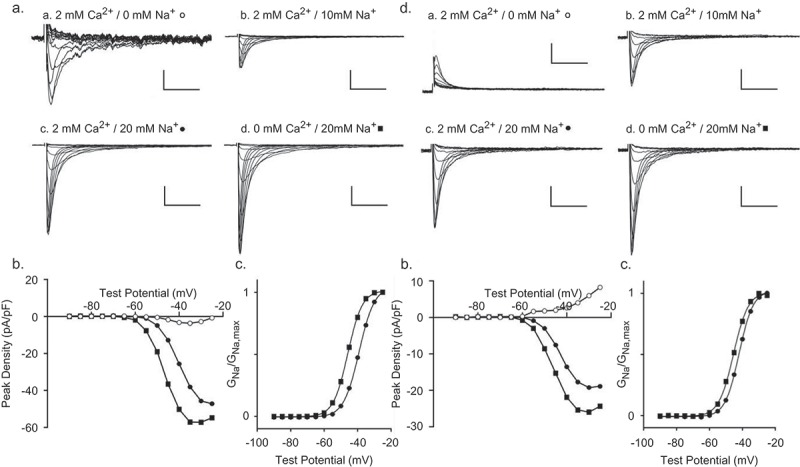
**Inward/outward currents were also recorded from tsA-201 cells expressing *SCN5A* (Nav1.5)**. Whole-cell currents, evoked in response to 300 ms voltage steps to test potentials between −90 and −25 mV (in 5 mV increments) from −100 mV and from −50 mV with 2 mm Ca^2+^/0 mm Na^+^ in the bath and 120 mm Cs^+^ (or K^+^) in pipettes, were recorded from visually identified eGFP-positive tsA-201 cells (n = 20) in cultures transfected with plasmids encoding *SCN5A* and *eGFP*, as described in **Methods**. The bath solution was then changed sequentially to one containing 2 mm Ca^2+^/10 mm Na^+^, 2 mm Ca^2+^/20 mm Na^+^ and 0 mm Ca^2+^/20 mm Na^+^ and the currents were again evoked (from both HPs). The current waveforms, obtained by off-line digital subtraction of the recordings evoked from −50 mV from those evoked from −100 mV, as described in the legend to Figure 5, under the different ionic conditions were analyzed. In 3 of the (20) Nav1.5-expressing tsA-201 cells, inward Ca^2+^ currents were recorded (*A*, panel a), whereas only outward currents were observed in the other 17 cells (*D*, panel a). (a) Increasing the Na^+^ concentration in the bath from 0 mm (panel a; o) to 10 mm (panel b) and 20 mm (panel c; ●), increased inward current amplitudes. Exchange of the extracellular solution to one containing 0 mm Ca^2+^ (replaced by 2 mm Mg^2+^) and 20 mm Na^+^ resulted in a further increase in the peak inward current amplitude (panel d; ■). Scale bars are 1 pA/pF and 10 ms in panel a and 10 pA/pF and 10 ms in panels b, c and d. Peak inward Ca^2+^ (o) and Na^+^ (●,■) currents for the cell in (*A*) are plotted in (b). The normalized voltage-dependences of activation of the currents recorded in 20 mm Na^+^ and 2 mm Ca^2+^ (●) and 20 mm Na^+^ and 0 Ca^2+^ (■) are plotted and fitted with single Boltzmanns (●: V_1/2_ = −39 mV, *k *= 4.6; ■: V_1/2_ = −46 mV, *k *= 4.3) in (c). (d) Representative outward currents recorded from an eGFP-positive, Nav1.5-expressing tsA-201 cell with 2 mm Ca^2+^/0 mm Na^+^ in the bath and 145 mm K^+^ in the pipette (panel a) are shown. Increasing the Na^+^ concentration to 10 mm (panel b) and 20 mm Na^+^ in the presence (panel c) and absence of 2 mm Ca^2+^ (panel d) revealed increasing inward Na^+^ currents. Scale bars are 5 pA/pF and 10 ms in panels a, b, c and d. Peak outward K^+^ (o), and inward Na^+^ (●,■) currents for the cell in (*D*) are plotted as a function of the test potential in (*E*). The normalized voltage-dependences of activation of the peak inward currents recorded in 20 mm Na^+^ and 2 mm Ca^2+^ (●) and 20 mm Na^+^ and 0 Ca^2+^ (■) are plotted and fitted with a single Boltzmann (●: V_1/2_ = −45 mV, *k *= 3.9; ■: V_1/2_ = −50 mV, *k *= 4.2) in (f).

In three (of the 20) eGFP-positive tsA-201 cells from which recordings were obtained using the protocols described above, inward currents were observed (Figure 6(a-c)), whereas in the other 17 cells, only outward Cs^+^ (K^+^) were detected (see below; Figure 6(d–f)). No inward or outward currents, however, were recorded from tsA-201 cells (n = 5) transfected with only the eGFP-expressing (i.e. without Nav1.5) plasmid (Supplemental Figure 1). Recordings with 0 Na^+^/2 mm Ca^2+^ in the bath from one of the Nav1.5-expressing tsA-201 cells in which inward currents were detected are presented in Figure 6(a) (panel a). Similar to the results obtained in mouse IVS myocytes (Figure 5), inward current amplitudes increased markedly (note the change in the scale) with 10 mm Na^+^ (and 2 mm Ca^2+^) in the bath (Figure 6(a), panel b), and further with 20 mm Na^+^ (and 2 mm Ca^2+^) in the bath (Figure 6(a), panel c). In addition, inward current amplitudes were further increased when the Ca^2+^ was removed (and replaced with 2 mm Mg^2+^) from the bath (Figure 6(a), panel d). The peak inward current-voltage relations for the recordings (in A) obtained with 2 mm Ca^2+^/0 mm Na^+^ (o), 2 mm Ca^2+^/20 mm Na^+^ (●) and 0 mm Ca^2+^/20 mm Na^+^ (■) in the bath are presented in Figure 6(b). Similar to the results obtained in mouse IVS myocytes, the inward currents followed monoexponential decay kinetics with 0 Na^+^ in the bath, but were best described by the sum of two exponentials with 20 mm Na^+^ (and either 2 mm Ca^2+^ or 0 mm Ca^2+^) in the bath (Supplemental Figure 2). Also, similar to the results in mouse IVS cells (Figure 5), the peak of the current-voltage curve (Figure 6(b)) and the normalized conductance versus voltage plot (Figure 6(c)) are shifted in the hyperpolarizing direction for recordings obtained with 0 mm Ca^2+^/20 mm Na^+^ (■), compared with 2 mm Ca^2+^/20 mm Na^+^ (●), in the bath.

In most (17/20 cells) tsA-201 cells expressing Nav1.5, only outward currents were observed in the presence of 2 mm Ca^2+^/0 mm Na^+^ in the bath and 145 mm K^+^ (Figure 6(d), panel a) or 120 mm Cs^+^ (not shown) in the recording pipettes. With increased Na^+^ (10 mm, 20 mm) in the bath, the outward currents progressively decreased and inward Na^+^ currents were recorded (Figure 6(d), panels b and c). Removal of the Ca^2+^ (replaced with Mg^2+^) from the bath further increased the inward current amplitudes (Figure 6(d), panel d). Peak outward K^+^ (o), and inward Na^+^ (●,■) currents are plotted as a function of test potential in Figure 6(e). The normalized conductance-voltage relations for the peak inward Na^+^ currents recorded in 20 mm Na^+^ and 2 mm Ca^2+^ (●) and 20 mm Na^+^ and 0 Ca^2+^ (■), fitted with single Boltzmanns, are presented in Figure 6(f). Similar to the findings in myocytes (Figure 3), the TTX-sensitive inward Ca^2+^ and outward Cs^+^/K^+^ currents in Nav1.5-expressing tsA-201 cells were blocked by MTSEA (data not shown).

### Differential expression of TTX-sensitive inward/outward currents in human LV myocytes

**Figure 7.**
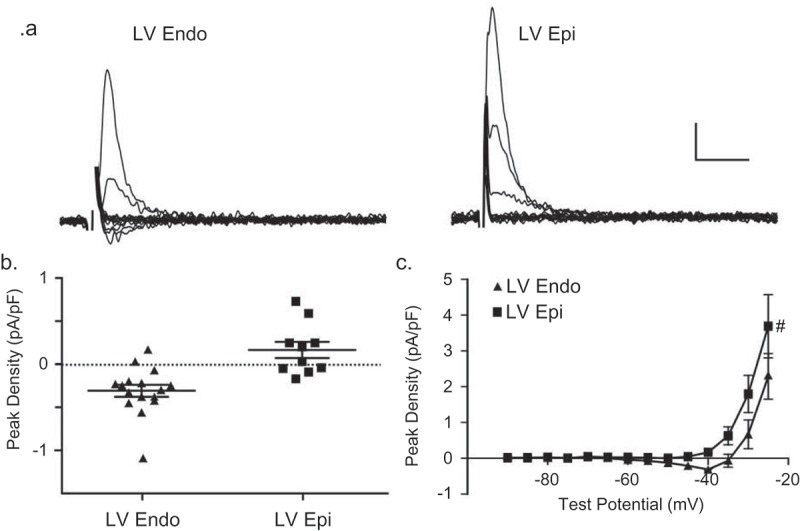
**TTX-sensitive currents in human ventricular myocytes**. With 2 mm Ca^2+^/0 mm Na^+^ in the bath and 120 mm Cs^+^ in pipette, inward currents were measured as described in legend to Figure 2 from human LV endocardial (LV Endo) and LV epicardial (LV Epi) myocytes before and after bath applications of 10 *µ*m TTX. (a) Representative (10 *µ*m) TTX-sensitive current waveforms; scale bars are 0.5 pA/pF and 10 ms. (b) Peak densities of the TTX-sensitive currents evoked at −40 mV from a HP of −100 mV in human LV Endo (●; n = 16) and LV Epi (■; n = 10) myocytes are plotted. (c) Mean ± SEM peak TTX-sensitive current densities in human LV Endo (●; n = 16) and LV Epi (■; n = 10) myocytes are plotted as a function of test potential; the densities of the currents in human LV endo and epi myocytes are significantly (^#^*P* < 0.05; two-way ANOVA) different.

Whole-cell recordings were obtained from myocytes isolated from the endocardial (LV Endo) and epicardial (LV Epi) surfaces of human LV free wall (see Methods) with 2 mm Ca^2+^ and 0 mm Na^+^ in the bath and 120 mm Cs^+^ in the recording pipettes. Currents, evoked from a HP of −100 mV in response to 300 ms test potentials between −90 mV to −25 mV (in 5 mV increments), were recorded in the absence and in the presence of 10 µM TTX, and the TTX-sensitive currents, obtained by offline digital subtractions of the records before and after exposure to TTX, were analyzed. As illust-rated in the representative records shown in Figure 7(a), small TTX-sensitive inward currents were observed in human LV Endo cells, whereas TTX-sensitive outward currents were seen in all human LV Endo (Figure 7(a), left) and LV Epi (Figure 7(a), right) myocytes. The peak TTX-sensitive current densities recorded in individual cells at a test potential of −40 mV from a HP of −100 mV in LV Endo (■; *n* = 16) and LV Epi (●; n = 10) are presented in Figure 7(b). The mean ± SEM peak TTX-sensitive current densities in human LV Endo (■; *n* = 16) and LV Epi (●; n = 10) myocytes are plotted as a function of test potential in Figure 7(c).

## Discussion

The experiments here identified TTX-sensitive inward Ca^2+^ currents and outward Cs^+^/K^+^ currents in myocytes isolated from adult mouse interventricular septum, LV apex, LV endocardial and LV endocardial myocytes, as well as in LV endocardial and epicardial myocytes isolated from non-failing human hearts, although marked differences in the relative amplitudes of the currents were observed in cells isolated from different regions of the LV in both mouse and human. The rapidly activating and inactivating, TTX-sensitive inward Ca^2+^ currents in adult mouse IVS myocytes were unaffected by Ni^2+^ and by verapamil (Figure1) revealing that neither T-type nor L-type Ca^2+^ channels contribute. In marked contrast, the rapidly activating and inactivating inward currents and the outward Cs^+^/K^+^ currents in mouse myocytes were blocked by MTSEA, a selective inhibitor of Nav1.5-encoded channels [19,34,35].

Additional experiments revealed that TTX-sensitive inward Ca^2+^ currents *and* outward Cs^+^/K^+^ currents were also observed in tsA-201 cells transiently transfected with a construct encoding *SCN5A* (Nav1.5), and that the pharmacological and the time- and voltage-dependent properties of the heterologously expressed Nav1.5-encoded currents were indistinguishable from those of native ventricular (TTX-sensitive inward and outward) currents in mouse and human ventricular myocytes. Also, similar to the findings in mouse ventricular myocytes, the TTX-sensitive inward Ca^2+^ and outward Cs^+^/K^+^ currents in Nav1.5-expressing tsA-201 cells were blocked by MTSEA. The simplest interpretation of these combined results is that *both* the inward Ca^2+^ currents *and* the outward Cs^+^/K^+^ currents recorded in mouse and human ventricular myocytes when extracellular Na^+^ is removed reflect currents through Nav1.5-encoded Na^+^ channels.

### Regional differences in the functional expression of TTX-sensitive myocardial currents

Marked regional differences were observed in the expression of TTX-sensitive inward Ca^2+^ and outward Cs^+^/K^+^ currents in adult mouse ventricles. *I*_Ca(TTX)_, for example, was identified in all myocytes isolated from the mouse IVS, although peak inward Ca^2+^ current amplitudes/densities were quite variable among (mouse IVS) cells. Heterogeneities in *I*_Ca**(TTX)**_ densities have also been reported in human atrial myocytes [4]. In addition, in ~40% of the mouse IVS cells studied, outward (Cs^+^) currents were also observed at the more positive test potentials. Heterogeneous expression of *I*_Ca(TTX)_ and TTX-sensitive outward (Cs^+^/K^+^) currents was also evident in mouse LV apex and LV free wall endocardial and epicardial myocytes, as well as in human LV endocardial and epicardial myocytes. These combined observations are quite different from the findings of Alvarez and colleagues [11] who reported no significant differences in the amplitudes/densities of ***I*_Ca(TTX)_** in different regions of infarcted rat left ventricles, suggesting species differences in the expression of TTX-sensitive currents. Alternatively, it is possible that the homogeneous expression of ***I*_Ca(TTX)_** reported by Alvarez and colleagues [11] reflects post-infarct remodeling, a hypothesis that warrants direct testing in mouse and human ventricles.

### Molecular determinants of TTX-sensitive inward Ca^2+^ and outward Cs^+^/k^+^ currents

The sensitivity to the membrane (holding) potential and to TTX led to suggestions that *I*_Ca(TTX)_ reflects inward Ca^2+^ flux through Nav channels encoded by the predominate Nav α subunit expressed in the heart, Nav1.5 [36]. The time- and voltage-dependent properties of *I*_Ca(TTX)_, however, are distinct from TTX-sensitive cardiac Nav currents [1,3,4,6]. In addition, TTX-sensitive cardiac Nav currents display greater ion-selectivity and lower permeability to Cs^+^ and/or K^+^ [9,10]. Interestingly, it was reported that an antisense oligonucleotide directed against rat Nav1.5, although resulting in a marked reduction in *I*_Na_ in adult rat ventricular myocytes, had no effect on *I*_Ca(TTX)_ [20], observations interpreted as suggesting a role(s) for novel (non Nav1.5-) Nav α subunit-encoded channels in the generation of *I*_Ca(TTX)_ and TTX-sensitive outward Cs^+^/K^+^ currents.

The experiments here, however, revealed TTX-sensitive inward Ca^2+^ currents *and* outward Cs^+^/K^+^ currents in tsA-201 cells transiently transfected with a cDNA construct encoding human *SCN5A* (Nav1.5). Similar to the observations in mouse and human ventricular myocytes, these experiments also revealed TTX-sensitive inward Ca^2+^ currents in a subset of the transiently transfected tsA-201 cells and outward Cs^+^/K^+^ currents in the others. The observed regional differences in the functional expression of TTX-sensitive inward Ca^2+^ currents and outward Cs^+^/K^+^ currents in mouse and human ventricular myocytes suggest that native Nav1.5-encoded cardiac Na^+^ channels are molecularly heterogeneous. Interestingly, it was recently reported that there are marked differences in *Scn5a* transcript expression levels and functional Nav current densities in adult mouse left and right ventricular endocardium and epicardium [37]. Cell type-specific differences in the densities/properties of TTX-sensitive, cardiac Nav1.5-encoded currents could also reflect the functional consequences of the differential splicing of *Scn5a*/*SCN5A* transcripts [38], as has been demonstrated for invertebrate Na^+^ and Ca^2+^ channels [39–41], as well as for the critical tight junction protein, claudin-10 [42,43] and TRPM3 [44]. Regional differences in the expression/functioning of Nav channel accessory subunits [45] and/or of other Nav channel regulatory proteins [46,47], as well as heterogeneities in post-translational modifications of Nav1.5 [48] and/or of one or more Nav channel accessory/regulatory proteins [45–47], could also play a role. Additional experiments are needed to define the molecular mechanisms underlying the observed regional differences in the functional expression and properties of native Nav1.5-encoded cardiac Na^+^ channels in mouse and human ventricles.

### Functional implications of heterogeneous expression of TTX-sensitive myocardial currents

The observed regional differences in the functional expression of TTX-sensitive inward Ca^2+^ currents and outward Cs^+^/K^+^ currents suggest that there are (at least) two molecularly distinct types of *Scn5a-*/*SCN5A-*encoded channels in both mouse and human LV myocytes. The molecular determinants of these channels may also be distinct from the Nav1.5-encoded channels that mediate inward Na^+^ flux and control cardiac myocyte action potential generation and propagation that display low Ca^2+^ and Cs^+^/K^+^ permeability [13,14]. Together with the observation that *I*_Ca(TTX)_ activates at potentials negative to the activation of I_Na_, these results suggest a possible role for Ca^2+^ entry through *I*_Ca(TTX)_ channels in regulating myocardial excitability and rhythmicity, particularly in the subthreshold range of membrane potentials. If there are changes in the relative expression and/or the distribution of *I*_Ca(TTX)_ channels under pathophysiological conditions [11], these could have functional consequences, perhaps contributing to the generation and maintenance of cardiac arrhythmias. Studies focused on defining the molecular basis of functional myocardial Nav1.5-encoded channel diversity and the mechanisms controlling the functional expression of these channels will be needed to explore these hypotheses directly.

## Supplementary material

Supplementary data for this article can be here.
