# Supplementary material for: Regional differences in the expression of tetrodotoxin-sensitive inward Ca2+ and outward Cs+/K+ currents in mouse and human ventricles
Source: Channels (Austin). 2019 Feb 1;13(1):72–87. doi: 10.1080/19336950.2019.1568146 (PMC6380286; doi:10.1080/19336950.2019.1568146)
Supplement: Supplemental Material [file kchl-13-01-1568146-s001.pptx]

## Slide 1
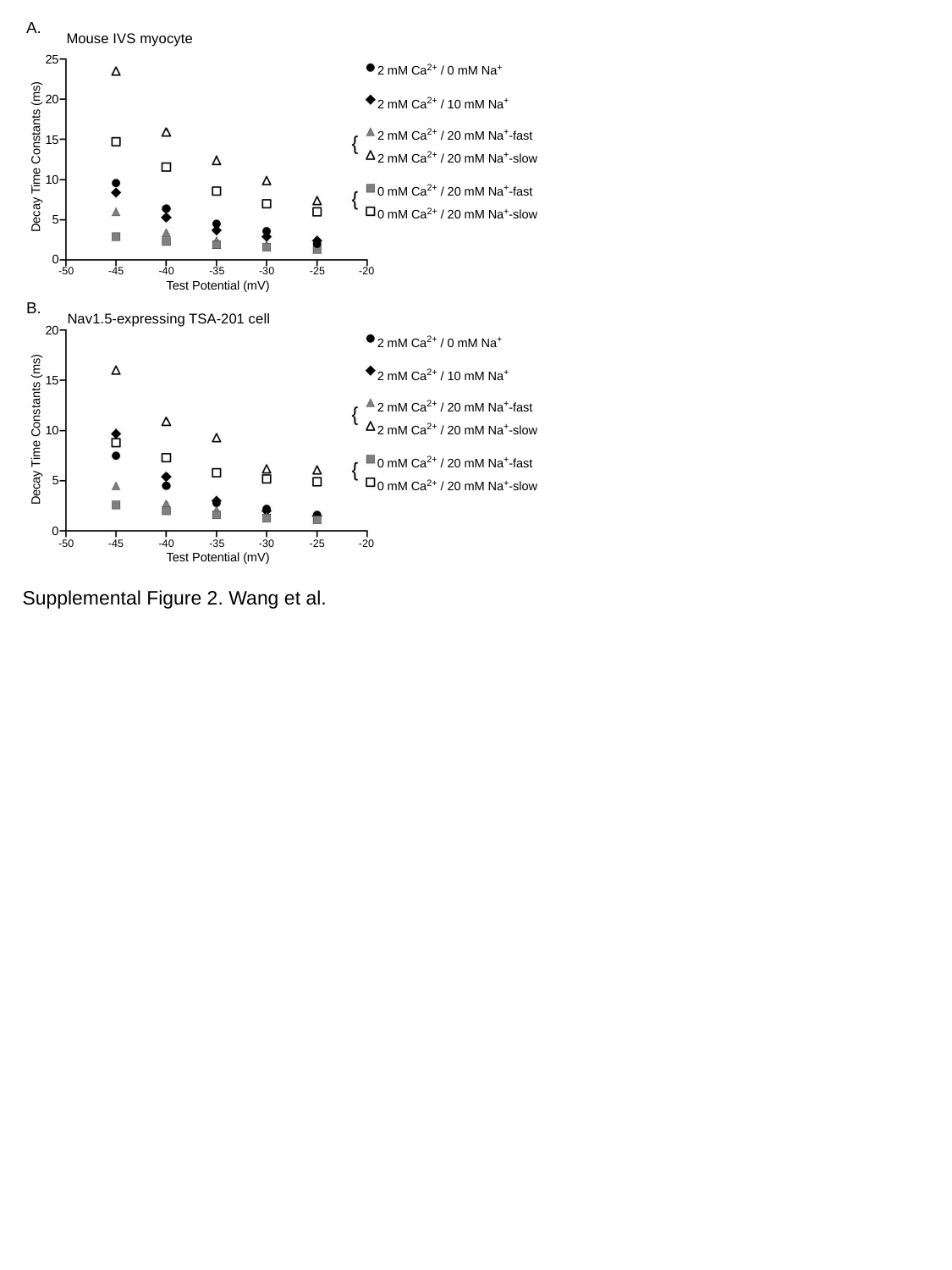

A.
Mouse IVS myocyte
25
2 mM Ca2+ / 0 mM Na+
20
2 mM Ca2+ / 10 mM Na+
2 mM Ca2+ / 20 mM Na+-fast
15
Decay Time Constants (ms)
2 mM Ca2+ / 20 mM Na+-slow
10
0 mM Ca2+ / 20 mM Na+-fast
0 mM Ca2+ / 20 mM Na+-slow
5
0
-50
-45
-40
-35
-30
-25
-20
Test Potential (mV)
B.
Nav1.5-expressing TSA-201 cell
20
2 mM Ca2+ / 0 mM Na+
2 mM Ca2+ / 10 mM Na+
15
2 mM Ca2+ / 20 mM Na+-fast
Decay Time Constants (ms)
2 mM Ca2+ / 20 mM Na+-slow
10
0 mM Ca2+ / 20 mM Na+-fast
5
0 mM Ca2+ / 20 mM Na+-slow
0
-50
-45
-40
-35
-30
-25
-20
Test Potential (mV)
Supplemental Figure 2. Wang et al.
