# Supplementary material for: Regional differences in the expression of tetrodotoxin-sensitive inward Ca2+ and outward Cs+/K+ currents in mouse and human ventricles
Source: Channels (Austin). 2019 Feb 1;13(1):72–87. doi: 10.1080/19336950.2019.1568146 (PMC6380286; doi:10.1080/19336950.2019.1568146)
Supplement: Supplemental Material [file kchl-13-01-1568146-s002.pptx]

## Slide 1
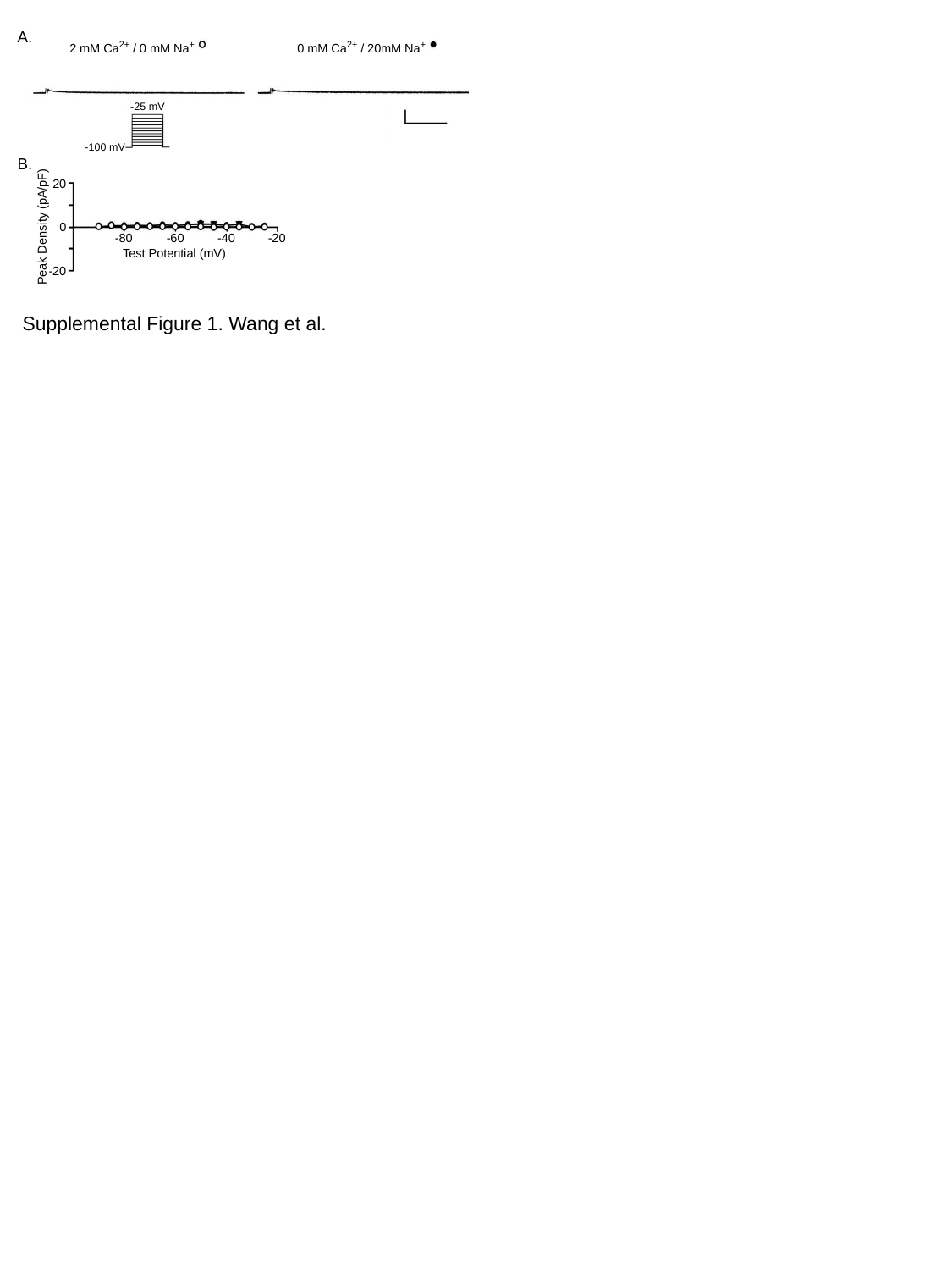

A.
2 mM Ca2+ / 0 mM Na+
0 mM Ca2+ / 20mM Na+
-25 mV
-100 mV
B.
20
Peak Density (pA/pF)
0
-80
-60
-40
-20
Test Potential (mV)
-20
Supplemental Figure 1. Wang et al.
